# Human iPSC-derived chondrocytes mimic juvenile chondrocyte function for the dual advantage of increased proliferation and resistance to IL-1β

**DOI:** 10.1186/s13287-017-0696-x

**Published:** 2017-11-02

**Authors:** Jieun Lee, Piera Smeriglio, Constance R. Chu, Nidhi Bhutani

**Affiliations:** 1 Department of Orthopaedic Surgery, Stanford University School of Medicine, 300 Pasteur Drive, Edwards Bldg., R164, Stanford, CA 94305-5341 USA; 2 Veterans Administration Palo Alto Health Care System, Palo Alto, CA USA

**Keywords:** Human iPSC-derived chondrocytes, Cartilage, Inflammation, Juvenile chondrocytes, CD24

## Abstract

**Background:**

Induced pluripotent stem cells (iPSC) provide an unlimited patient-specific cell source for regenerative medicine. Adult cells have had limited success in cartilage repair, but juvenile chondrocytes (from donors younger than 13 years of age) have been identified to generate superior cartilage. With this perspective, the aim of these studies was to compare the human iPSC-derived chondrocytes (hiChondrocytes) to adult and juvenile chondrocytes and identify common molecular factors that govern their function.

**Methods:**

Phenotypic and functional characteristics of hiChondrocytes were compared to juvenile and adult chondrocytes. Analyses of global gene expression profiling, independent gene expression, and loss-of-function studies were utilized to test molecular factors having a regulatory effect on hiChondrocytes and juvenile chondrocyte function.

**Results:**

Here, we report that the iPSC-derived chondrocytes mimic juvenile chondrocytes in faster cell proliferation and resistance to IL-1β compared to adult chondrocytes. Whole genome transcriptome analyses revealed unique ECM factors and immune response pathways to be enriched in both juvenile and iPSC-derived chondrocytes as compared to adult chondrocytes. Loss-of-function studies demonstrated that CD24, a cell surface receptor enriched in both juvenile chondrocytes and hiChondrocytes, is a regulatory factor in both faster proliferation and resistance to proinflammatory cues in these chondrocyte populations.

**Conclusions:**

Our studies identify that hiChondrocytes mimic juvenile chondrocytes for the dual advantage of faster proliferation and a reduced response to the inflammatory cytokine IL-1β. While developmental immaturity of iPSC-derived cells can be a challenge for tissues like muscle and brain, our studies demonstrate that it is advantageous for a tissue like cartilage that has limited regenerative ability in adulthood.

**Electronic supplementary material:**

The online version of this article (doi:10.1186/s13287-017-0696-x) contains supplementary material, which is available to authorized users.

## Background

Human articular cartilage regeneration is inherently inefficient [1]; hence cartilage injuries persist even in young and healthy adults, require intermittent medical attention, and are increasingly translated to early onset of osteoarthritis. No effective treatments exist besides pain management or eventual total joint replacement, constituting a substantial medical burden on the US economy. Clinically approved cell-based approaches to treat cartilage defects utilize implantation of autologous chondrocytes or stimulation of endogenous mesenchymal stem cells (MSC) through microfracture [2]; however, these approaches suffer from limited cell availability as well as generation of functionally inferior fibrocartilage.

Unlike adults, pediatric populations can sometimes show effective cartilage repair. In basic science studies, superior repair was observed in young rabbits as compared to adults [3], and even a developmental in-utero cartilage defect in lambs showed efficient repair [4]. Recent reports have suggested that juvenile chondrocytes from donors younger than 13 years old have a potential for superior cartilage regeneration compared to adult chondrocytes [5–7]. Small cohort studies (nine patients) in a 2-year timeframe have shown clinical benefit upon allogeneic juvenile cartilage transplantation in focal cartilage defects or injuries in the knee [8]. Other studies have reported additional advantages of juvenile chondrocytes in that they are immune privileged [6], can generate cartilage in the absence of chondrocyte hypertrophy [5, 7], and provide inductive cues for MSCs leading to generation of hyaline cartilage [9]. However, a major challenge to the widespread clinical use of juvenile cartilage or chondrocytes is the limited donor availability.

Multiple recent studies have focused on developing methods to utilize ‘reprogramming’ approaches and human induced pluripotent stem cells (hiPSCs) as a potentially limitless patient-autologous cell source for generation of chondrocytes [10–13]. In addition, the iPSC technology provides an attractive tool for modeling skeletal diseases-in-a-dish since patient blood or skin cells can be used to generate cartilage through reprogramming, allowing the study of the disease pathology as well as drug screening on the reprogrammed cells [14, 15]. It has been well established for multiple tissue types, notably for heart cells [16] and neurons [17], that hiPSC-derived cells show a neonatal phenotype lacking developmental maturity. We aimed to harness the potential developmental immaturity of the hiPSC-derived chondrocytes (hiChondrocytes) and to test whether these hiChondrocytes would mimic the superior regenerative properties of juvenile chondrocytes. Toward this goal, we recently developed a growth factor-based method that is relatively quick (2 weeks) and highly efficient in directing hiPSC differentiation to chondrocytes (hiChondrocytes) [18]. Our group and others previously reported striking phenotypic and functional differences between juvenile and adult human chondrocytes [19, 20], including increased proliferation and ECM production by the juvenile chondrocytes compared to adult chondrocytes [20]. In addition, we have recently reported distinct global gene expression profiles for juvenile and adult chondrocytes and identified differential molecular regulators and pathways [21], making it feasible to study the molecular as well as functional similarities and differences between juvenile and hiPSC-derived chondrocytes.

In this report, we have analyzed phenotypic and functional characteristics of hiChondrocytes compared to juvenile and adult chondrocytes, in conjunction with global gene expression profiling and loss-of-function studies, to understand the molecular basis for their differential phenotypes. hiChondrocytes and juvenile chondrocytes reveal a dual advantage in having a higher proliferative potential as well as resistance to inflammatory cytokine IL-1β. These phenotypic similarities are paralleled by a common set of molecular factors that are enriched in both juvenile chondrocytes and hiChondrocytes compared to adult chondrocytes. CD24 is one such beneficial cell-surface factor and its loss leads to a reduction in both cell proliferation and resistance to IL-1β. These studies therefore provide mechanistic insights regarding the proliferative potential of hiPSC-derived chondrocytes and elucidate novel molecular factors that can be exploited for devising new cellular or small molecule-based therapies for augmenting cartilage tissue generation.

## Methods

### Chondrocyte isolation and culture

As described previously [21], juvenile articular chondrocytes at 24 weeks (fetus; J1), 6 months (J2), and 18 months (J3) were purchased from Lonza (Clonetics™; Lonza Walkersville, Inc.) and cultured in Chondrocyte Growth Medium (Clonetics™; Lonza Walkersville, Inc.). Human adult articular chondrocytes (35-year-old (A1), 27-year-old (A2), 34-year-old (A3), and 39-year-old (A4) females) were harvested from grossly normal cartilage pieces that were discarded during notchplasty or debridement from non-OA patients under protocols approved by Stanford University’s human subjects Institutional Review Board (IRB). Chondrocytes were cultured in Chondrocyte Growth Medium (Clonetics™; Lonza Walkersville, Inc.) in high-density monolayers for limited passages (2–4), as described previously [20, 21]. Human iPSC-derived chondrocytes (hiChondrocytes) that we have characterized previously [18] (five different independently reprogrammed batches) were cultured in Chondrocyte Growth Medium (Clonetics™; Lonza Walkersville, Inc.) up to passage 4.

### Microarray analyses

For microarray expression analyses, purified RNA was run on a Human gene 1.0 ST array (Affymetrix). Three independent samples for hiChondrocytes, juvenile chondrocytes, and adult chondrocytes were run on Human Gene 1.0 ST Arrays (Affymetrix). Data analysis was performed using the dChip software [22]. Normalization, comparison of gene expression values, filtering of significant expression probes, and clustering analysis of expression values were done within dChip as described by the manual. Network analysis of differentially expressed genes was performed using MetaCore (Thomson Reuters).

### IL-1β treatment

Chondrocytes (5 × 10^5^ cells) were cultured for 24 hours and then treated with control or IL-1β (10 ng/ml) in complete media for 48 hours prior to analyses.

### Single-cell Annexin V analyses

Apoptosis was measured by flow cytometry using a FITC-conjugated Annexin V and propidium iodide (PI) apoptosis kit (Invitrogen) according to the manufacturers’ instructions. Briefly, cells were seeded in sex-well plates at a density of 10^6^ cells/ml. After incubating cells for 24 hours, cells were harvested, washed with cold PBS, and incubated for 15 min with FITC-conjugated Annexin V to stain apoptotic cells and PI to stain necrotic cells. Fluorescence-labeled cells were measured by flow cytometry using a LSR II flow cytometer and analyzed with Flowjo software. Annexin V-positive cells were evaluated as apoptotic cells and Annexin V + PI cells as necrotic cells.

### Lentiviral preparation

293FT cells (6 × 10^6^ cells) were plated in T225 flasks and, after overnight incubation, transfected with 7.5 μg of VSV-G, 5.7 μg of TAT, 7.5 μg of Rev, 30 μg of Gag/Pol, and 15 μg of shCD24 lentiviral plasmid (Sigma) using Lipofectamine. After 48 hours, supernatant was collected, filtered through a 0.45-μm filter, and centrifuged at 17,100 rpm for 2 hours and 20 min. For CD24 knockdown, virus-containing supernatant was supplemented with 8 μg/ml polybrene and used at a 100 multiplicity of infection (MOI) to infect 5 × 10^4^ chondrocytes.

### Chondrocyte transplantation and cartilage generation in mice

Our previously reported method was used to synthesize chondroitin sulfate methacrylate (CS-MA). For chondrocyte encapsulation, 15 × 10^6^ chondrocytes/ml were suspended in a hydrogel solution consisting of 5% weight/volume (w/v) poly(ethylene glycol diacrylate) (MW 5000 g/mol), 3% w/v CS-MA, and 0.05% w/v photoinitiator (Irgacure D 2959; Ciba Specialty Chemicals, Basel, Switzerland) in medium. Chondrocyte-laden hydrogels, 5 mm in diameter and 2.5 mm in height, were initially cultured in vitro for 24 hours and subsequently engrafted subcutaneously in NCr-Foxn1nu-immunodeficient mice (Taconic, Oxnard, CA, USA). All samples were harvested after 28 days for further analyses. All animal procedures were carried out under the approval of the Stanford University Administrative Panel on Laboratory Animal Care (APLAC).

#### Immunohistochemistry

For immunostaining of cells in monolayers, cells were fixed in 4% paraformaldehyde (Sigma, St. Louis, MO, USA) for 10 min and then permeabilized with cold 0.4% Triton X-100 (Sigma) in PBS for 15 min. The cells were then incubated for 1 hour in PBS containing 1% BSA–10% FBS–0.4% Triton X-100 (for blocking). For 3D culture staining, hydrogels were fixed in 4% paraformaldehyde overnight, transferred to 70% ethanol, and included in paraffin (Histoserv Inc.). Sections were then deparaffinized through xylene and graded alcohols. A step of enzymatic antigen retrieval with 0.1% Trypsin in PBS was performed prior to blocking (2% goat serum–3% BSA–0.1% Triton X-100) for 1 hour.

After the blocking step, antibodies against Aggrecan (1:500), Col2a1 (1:100; Abcam), Col10 (1:100; Abcam), Sox9 (1:100; Santa Cruz), or anti-human Ki67 (1:500; ebiosciences) were added overnight in blocking buffer. The following day, cells were washed three times with PBS and then incubated for 1 hour at room temperature with Alexa 594-conjugated goat anti-rabbit secondary antibody (1:250; Gibco, Invitrogen). Cellular DNA was counterstained with DAPI (Life Technologies, Carlsbad, CA, USA). The antibody anti-AGC used for this study was a kind gift from Prof. R.L. Smith [23].

#### Biochemical analyses

Cell-hydrogel constructs (*n* = 4) were weighed wet, lyophilized, weighed dry, and digested in papainase solution (Worthington Biochemical, Lakewood, NJ, USA) at 60 °C for 16 hours. The DNA content was measured using the PicoGreen assay (Molecular Probes, Eugene, OR, USA) with Lambda phage DNA as standard. The sulfated glycosaminoglycan (sGAG) content was quantified using the 1,9-dimethylmethylene blue (DMMB) dye-binding assay with shark chondroitin sulfate (Sigma) as standard [24]. The GAG content of the acellular hydrogels was determined as a negative control, since it is included in the total amount of GAG released by the encapsulated cells during the 3–6 weeks of culture.

### Statistical analyses

Data are reported as the mean ± standard error of the mean (SEM). Statistical significance of data was determined by applying a two-tailed Student’s *t* test and *p* < 0.01 is reported as significant. For multiple comparisons, statistical analyses were performed using one-way ANOVA followed by Bonferroni’s correction and *p* < 0.01 is reported as significant.

## Results

### Comparative global gene expression patterns in hiChondrocytes, juvenile chondrocytes, and adult chondrocytes

We aimed to firstly characterize hiChondrocytes in terms of their proliferation and chondrogenic gene expression with reference to ‘juvenile’ and ‘adult’ chondrocytes. The hiChondrocytes were differentiated from human induced pluripotent stem cells (hiPSCs) using a step-wise protocol with growth factors defined by us previously (Additional file 1: Figure S1a) [18]. The isolation, maintenance, and characterization of the juvenile and adult chondrocytes has been described previously [20, 21] and summarized in Methods. Upon testing the proliferation rates of hiChondrocytes compared to juvenile and adult chondrocytes, the doubling time of hiChondrocytes was found to be 35–36 hours and was very similar to the doubling time of juvenile chondrocytes (35–37 hours) (Additional file 1: Figure S1b). The doubling time for adult chondrocytes, however, was much slower at 45–46 hours (Additional file 1: Figure S1b). These observations are consistent with previous reports that juvenile chondrocytes proliferate faster than adult chondrocytes [5]. We observe that the hiChondrocytes mimic the faster proliferation rate of juvenile chondrocytes.

To investigate molecular signatures of hiChondrocytes comparative to juvenile and adult chondrocytes, we utilized exon microarrays to determine their global gene expression profiles using three independently differentiated hiChondrocyte lines (iC1–iC3). hiChondrocytes were cultured for limited passages and expanded in monolayer culture (1–2 passages in 2–4 days) before total RNA was extracted. Human Gene 1.0 ST Arrays (Affymetrix) were utilized and data analysis was performed using dChip as described previously [22] (Additional file 1: Figure S1c). Juvenile (JC1–JC3) and adult (AC1–AC3) chondrocyte profiles had been reported earlier [21] and were used as reference. Gene expression profiles of the hiChondrocytes (hiC1–hiC3) were very different from the hiPSCs. Correlation matrices generated for the global gene expression profiles for all of the samples showed a clustering of all chondrocyte samples such that the juvenile, adult, or hiChondrocyte populations could not be distinguished from each other while being distinct from iPSCs (Additional file 1: Figure S2). We have reported previously that the hiChondrocytes clustered with the adult chondrocytes in similar analyses [18]. Consistent with that analysis, globally the hiChondrocytes, adult chondrocytes, and juvenile chondrocytes are indistinguishable. While the hiC1 showed a higher correlation with the adult chondrocyte samples A1 and A2 compared to the juvenile samples J1 and J3, the hiC2 showed a similar correlation with A1, A2, J1, and J3 (Additional file 1: Figure S2). Inclusion of J2 and A3 further highlighted the variability between the donors, both adult and juvenile, showing that at the global level it is not possible to distinguish hiChondrocytes from juvenile or adult chondrocytes.

**Fig. 1:**
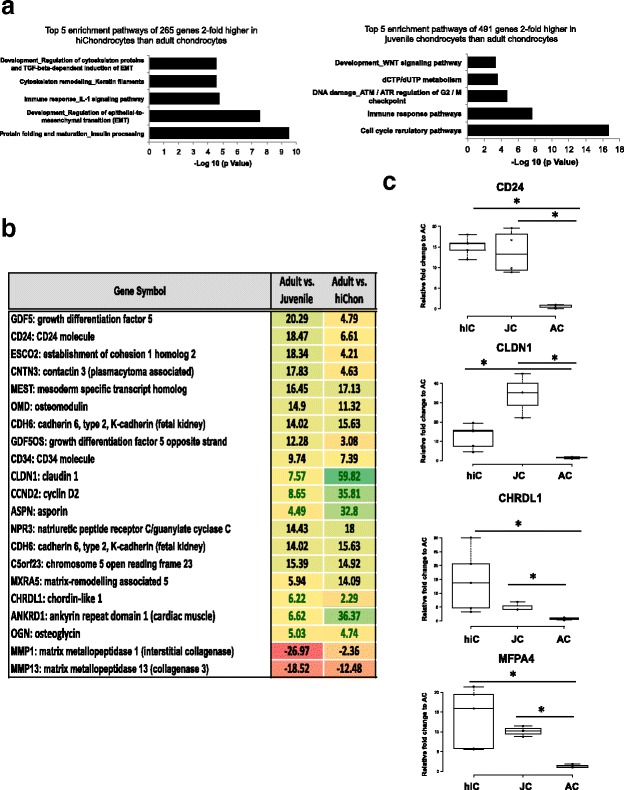
Common genes enriched in hiChondrocytes and juvenile chondrocytes include distinct ECM factors and the cell-surface marker *CD24*. (**a**) The  top five enriched pathways upon network analyses of genes were found to be 2-fold or higher in expression upon comparison with adult chondrocytes in hiChondrocytes (265 total genes) and in juvenile chondrocytes (491 genes). (**b**) The top 20 genes out of a total of 71 common genes found to be enriched in both hiChondrocytes and juvenile chondrocytes when compared to the adult chondrocytes. Array data consist of three independent donors for each chondrocyte type. (**c**) Real-time PCR shows gene expression levels of *CD24, CHRDL1, CLDN1, *and *MFAP4* in hiChondrocyte and juvenile chondrocyte samples relative to adult chondrocytes. Data represent five independent hiChondrocyte samples and four donors each for juvenile and adult chondrocytes. AC (adult chondrocytes), hiC human induced pluripotent stem cell-derived chondrocytes (hiChondrocytes), JC (juvenile chondrocytes)

Although the overall global gene expression patterns of the chondrocyte populations were similar, we wanted to explore whether there was a smaller subset of genes that were differentially enriched in the hiChondrocytes and juvenile chondrocytes. In order to identify such common molecular factors, differentially expressed genes in the hiChondrocytes and juvenile chondrocytes were both compared individually to the adult chondrocytes and ranked according to the average fold-change in transcript expression. Analysis of genes with a 2-fold or greater increase in expression identified 265 genes that were upregulated in the hiChondrocyte compared to the adult chondrocytes. In a similar analysis, 491 genes showed 2-fold or greater upregulation in the juvenile chondrocytes compared to the adult chondrocytes. Interestingly, network analyses on the genes with a 2-fold or greater increase in expression in hiChondrocytes or juvenile chondrocytes compared to adult chondrocytes identified both common and distinct pathways (Fig. 1a). The top five pathways enriched in the hiChondrocytes and juvenile chondrocytes included early chondrocyte development pathways, especially TGF-β-dependent pathways in hiChondrocytes and the Wnt signaling pathway in the juvenile chondrocytes. Surprisingly, related immune response pathways were differentially enriched both in hiChondrocytes and in juvenile chondrocytes (Fig. 1a).

Next, we intersected the 2-fold enriched genes in hiChondrocytes and juvenile chondrocytes and found 71 common genes. Upon focusing on the top 20 of these genes, we observed that these common genes were enriched to a different extent when compared to the adult chondrocytes (Fig. 1b). For example, while *GDF5* was 20-fold enriched in juvenile chondrocytes, it was only 5-fold increased in hiChondrocytes; similarly, the cell-surface marker *CD24* was 18-fold higher in juvenile chondrocytes but only 7-fold higher in hiChondrocytes compared to adult chondrocytes. A list of genes higher in hiChondrocytes or juvenile chondrocytes is presented in Additional file 1: Figure S3. Interestingly, the list of common enriched genes included several ECM genes that we had identified previously to be uniquely enriched in juvenile chondrocytes such as chordin-like 1 (*CHRDL1*) and microfibrillar associated protein 4 (*MFAP4*) [21]. Out of these genes, we had identified *CHRDL1* to have the ability to enhance proliferation of adult MSCs [21]. The gene list also included *CYCLIN D2* that can potentially account for the greater proliferative capacity of both hiChondrocytes and juvenile chondrocytes. Overall, these analyses suggested that hiChondrocytes expressed several of the key molecular factors that defined the juvenile chondrocyte fate. These observations were confirmed using quantitative real-time PCR for various key genes. It was observed by real-time PCR that *CD24, CHRDL1, CLDN1,* and *MFAP4 *were enriched in both hiChondrocytes and juvenile chondrocytes compared to adult chondrocytes (Fig. 1c). Some of the other genes were validated to be differentially expressed in hiChondrocytes and juvenile chondrocytes (*LBD2, EPHA5, ASPN, CDH10, NEDD9,* and *CD34*), demonstrating that the array results were of high quality and reproducible with an independent method of assessment (Additional file 1: Figure S4). Upon validating the gene expression of chondrocyte-related genes by real-time PCR, we found that the gene expression of *SOX9, COL2A*, and *ACAN* in hiChondrocytes is comparable to juvenile as well as adult chondrocytes (Additional file 1: Figure S5).

### Resistance to IL-1β in hiChondrocytes and juvenile chondrocytes compared to adult chondrocytes

**Fig. 2:**
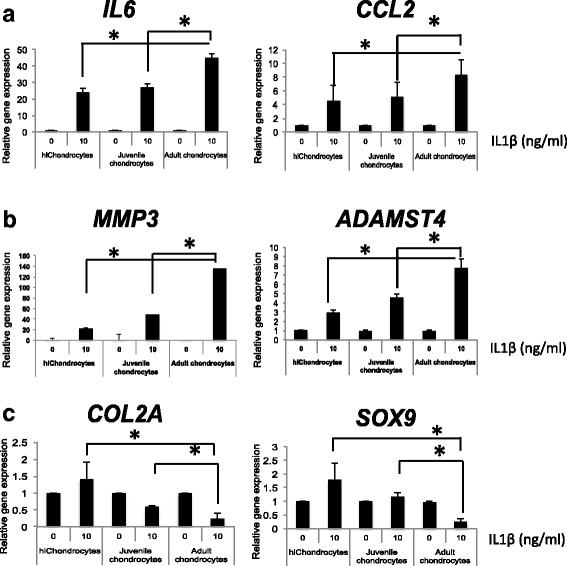
Immune resistance in hiChondrocytes and juvenile chondrocytes compared to adult chondrocytes. Upon IL-1β stimulation (10 ng/ml) gene expression of (**a**) inflammatory genes (*IL6* and *CCL2*), (**b**) catabolic genes (*MMP3* and *ADAMTS4*), and (**c**) chondrocyte regulatory genes (*COL2A* and *SOX9*) in hiChondrocytes, juvenile chondrocytes, and adult chondrocytes. *p < 0.01. Gene expression relative to control in the absence of IL-1β for each respective gene. Data represent two independent donors for each chondrocyte type and three replicate experiments

We have recently reported that cluster of differentiation 24 (CD24), which has previously been shown to modulate innate immunity [25], regulates differential response of juvenile and adult chondrocytes to inflammatory cues in an NF-κB-dependent manner [26]. Since CD24 is enriched in hiChondrocytes similar to juvenile chondrocytes, we hypothesized that hiChondrocytes will be resistant to inflammation similar to juvenile chondrocytes. For further functional studies, we utilized two representative donors from each chondrocyte population (J1 and J2, A1 and A2, hiC1 and hiC2) since these assays require a larger number of cells and were performed in triplicate. Chondrocytes were treated with IL-1β (0 or 10 ng/ml dosage) for 48 hours, and tested for the expression of inflammatory genes (*CCL2* and *IL6*) (Fig. 2). A significantly lower upregulation of both *CCL2* and *IL6* was observed in hiChondrocytes compared to adult chondrocytes (Fig. 2a). Similarly, upon testing a few catabolic genes implicated in osteoarthritis, we observed a significantly lower upregulation of *MMP3* and *ADAMTS4* in the hiChondrocytes and juvenile chondrocytes (Fig. 2b). hiChondrocytes and juvenile chondrocytes also maintained the chondrogenic gene expression (*COL2A* and *SOX9*) that was significantly decreased in the adult chondrocytes in response to IL-1β treatment (Fig. 2c). No change in Col1a1 was observed upon IL-1β treatment in either of the chondrocyte populations. Overall, the hiChondrocytes resembled the juvenile chondrocytes in a reduced response to inflammatory cytokines as compared to adult chondrocytes, showing that hiChondrocytes share the advantage of immune resistance with juvenile chondrocytes and should be able to maintain cartilage integrity better by resisting the conversion to fibrocartilage as well as upregulation of catabolic pathways.

### CD24 is a regulator for both resistance to IL-1β and higher proliferation in hiChondrocytes and juvenile chondrocytes

**Fig. 3:**
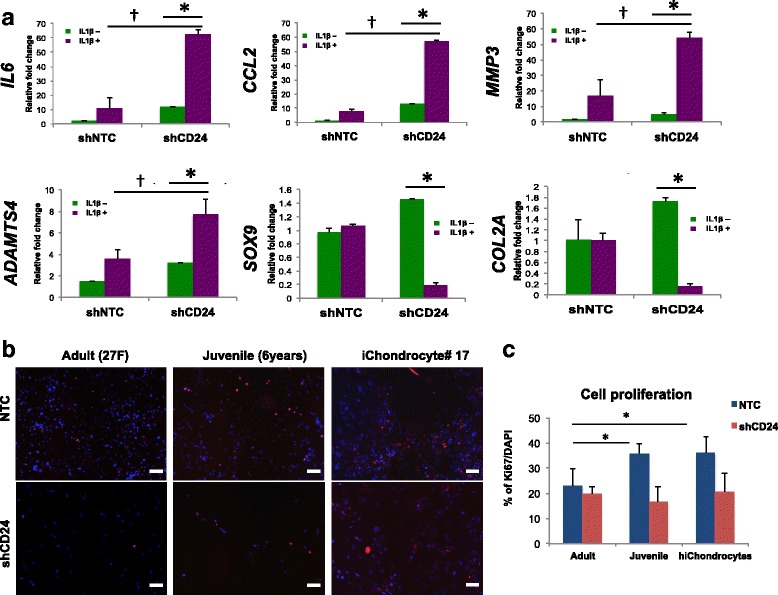
CD24 is a regulator for both resistance to IL-1β and higher proliferation in hiChondrocytes. (**a**) Loss of CD24 in hiChondrocytes increases the inflammatory response and dedifferentiation in the presence of IL-1β (10 ng/ml). In the absence of CD24 (shCD24), gene expression for *IL6, CCL2, MMP3, *and *ADAMTS4 *increases but decreases for the chondrogenic markers *SOX9* and *COL2A* in the absence and presence of IL-1β. *, † both *p* < 0.01. (**b**) Loss of CD24 reduces Ki67 expression in proliferating chondrocytes. Representative immunofluorescence images for Ki67 (red) expression in chondrocytes, in the presence (nontarget control (NTC)) or absence of CD24 (shCD24). Nuclei (blue) are stained with DAPI. Scale bar, 100 μm. (**c**) Quantification of percentage of Ki67^+^ cells (average of 10 fields). Ki67^+^/total cell ratio in the presence (NTC) or absence (shCD24) of CD24 in adult chondrocytes, juvenile chondrocytes, and hiChondrocytes. Data represent two independent donors for each chondrocyte type and three replicate experiments

We next tested whether CD24 regulates the resistance to inflammation in hiChondrocytes similar to juvenile chondrocytes as we have reported recently [26]. We utilized the previously validated shRNA against CD24 for knockdown at gene and protein levels, and compared it to a nontarget control shRNA. Real-time quantitative PCR and single-cell FACS analyses were used to confirm CD24 knockdown that was greater than 90% in all experiments (Additional file 1: Figure S6). Upon causing shRNA-mediated loss of CD24 in hiChondrocytes, we observed an upregulation of the inflammatory genes *IL6* and *CCL2* and catabolic genes *MMP3* and *ADAMTS4*, while the chondrogenic genes *COL2A* and *SOX9* remained unchanged (Fig. 3a). Next, hiChondrocytes transduced with either the nontarget control (shNTC) or CD24-specific shRNA (shCD24) were treated with IL-1β treatment (0 or 10 ng/ml dosage) for 48 hours. As expected, loss of CD24 in combination with IL-1β showed a significantly higher upregulation of *IL6*, *CCL2*, *MMP3*, as well as *ADAMTS4*, demonstrating that, similar to juvenile chondrocytes, CD24 renders resistance to IL-1β in hiChondrocytes (Fig. 3a).

We also noted during the course of experiments that loss of CD24 led to a modest decrease in cell numbers in all chondrocyte populations (Fig. 3b). Upon utilizing DAPI to quantitate cell numbers and Ki67 staining to mark actively proliferating cells 72 hours after chondrocyte transduction with a control or CD24 shRNA, the percentage of actively dividing cells was decreased from 30–35% cells to 15–18% cells in the absence of CD24 (Fig. 3c). Additionally, we wanted to test whether a loss of CD24 increased cell death. Annexin V expression at a single cell level by FACS was utilized as a measure of cells undergoing apoptosis in the presence or absence of CD24. There was no significant change in Annexin V expression upon loss of CD24 in all chondrocyte populations (Additional file 1: Figure S7), demonstrating that the decreased proliferation was not due to an increase in cellular apoptosis. These observations showed that CD24 enrichment in both juvenile chondrocytes and hiChondrocytes contributes to the dual advantage of higher proliferation and resistance to IL-1β.

### Comparative potential of hiChondrocytes and juvenile chondrocytes to generate cartilage in vivo in PEG-CS hydrogels

**Fig. 4:**
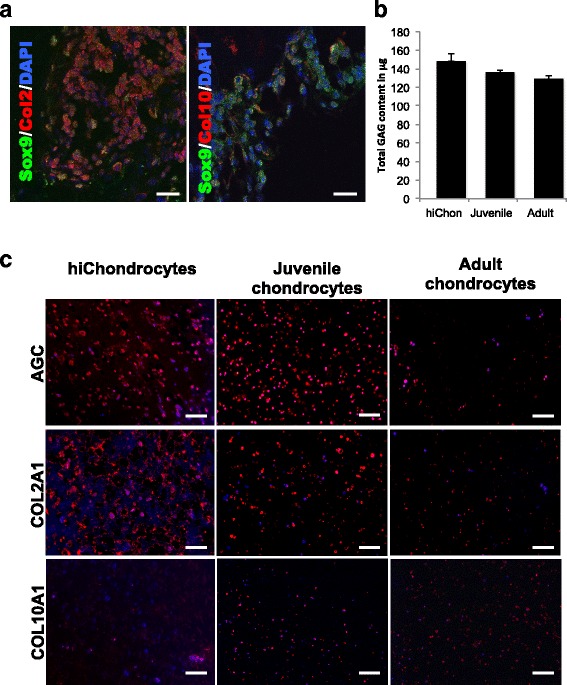
hiChondrocytes mimic juvenile chondrocytes in generation of superior cartilage in PEG-CS hydrogels in vivo. (**a**) Confocal microscopy for representative immunofluorescence staining for chondrocyte markers Sox9, Col2a1, and Col10a1 in hydrogel-capsulated hiChondrocytes cultured for 4 weeks in vivo. Scale bar, 50 μm. (**b**) Quantification of GAG production in hiChondrocyte-derived, juvenile chondrocyte-derived, and adult chondrocyte-derived cartilage constructs in vivo. The GAG content of the control acellular hydrogel was 60 μg. (**c**) Representative immunofluorescence staining for aggrecan, Col2a1, and Col10a1 in hydrogels containing hiChondrocytes, juvenile chondrocytes, and adult chondrocytes that were transplanted in mice and harvested after 4 weeks. Scale bar, 100 μm. Data represent two independent donors for each chondrocyte type and three replicate experiments. GAG glycosaminoglycan

It has been suggested that juvenile chondrocytes have the potential to generate superior hyaline-like cartilage in the absence of hypertrophy as compared to adult chondrocytes. The donor scarcity of juvenile cartilage can be overcome if hiChondrocytes show a similar capability to engineer hyaline-like cartilage. To assess the chondrogenic potential of hiChondrocytes comparative to juvenile and adult chondrocytes, we utilized a cartilage biomimetic scaffold that we have characterized previously to preserve juvenile chondrocyte function [20]. These biomimetic scaffolds also support cartilage generation by MSCs and ADSCs [27–29]. All chondrocytes—adult, juvenile, and hiChondrocytes—were encapsulated in chondroitin sulfate containing polyethylene glycol (CS-PEG)-based hydrogels as described previously, cultured for 24 hours in vitro, and engrafted subcutaneously in NCr-Foxn1nu-immunodeficient mice. The chondrocyte engrafted hydrogels, 5 mm in diameter and 2.5 mm in height, were harvested from the mice after 28 days and cartilaginous tissue formation was assessed both histologically and biochemically. Using antibodies specific to Sox9, Col2a1, and Col10a1, it was observed that cartilage pellets generated by hiChondrocytes showed abundant staining for Sox9 and col2a1 while col10a1 was almost negligible, showing an absence of hypertrophy (Fig. 4a). Extracellular matrix components produced were assayed using biochemical quantification of GAG as well as immunohistochemical staining for Aggrecan, and different types of collagens (Col2a1 and Col10a1) for cartilage constructs formed by hiChondrocytes, juvenile chondrocytes, and adult chondrocytes. Quantification of GAG production from the cartilage constructs generated by hiChondrocytes demonstrated only a modestly higher amount of GAG production compared to the constructs generated by juvenile and adult chondrocytes (Fig. 4b). However, both Aggrecan and cartilage-specific type II collagen (Col2a1) were more abundantly present throughout the cartilage constructs formed by hiChondrocytes and juvenile chondrocytes as compared to adult chondrocytes (Fig. 4c). Additionally, both hiChondrocytes and juvenile chondrocytes presented little staining for type X collagen (Col10a1) in hydrogels showing a lack of characteristic hypertrophic markers (Fig. 4c).

## Discussion

The central question for engineering a native-like tissue is firstly to identify and define the ‘ideal’ stem or progenitor cell type for tissue regeneration, and secondly the ‘cell source’ that can be used to derive such a progenitor cell. Although the tissue-resident stem cell-mediated regenerative processes are well defined and understood in molecular details for tissues like muscle, skin, and bone, cartilage regeneration or lack thereof remains enigmatic. Multiple studies have explored the presence of a stem/progenitor-like population in cartilage [30] and there is some recent evidence for the existence of small stem-like populations marked by growth differentiation factor 5 (Gdf5) [31] or lubricin (PRG4) [32]. However, it remains a clinical reality that these cell populations are unable to effectively regenerate into functional cartilage, unlike bone or muscle [1]. Because of the lack of a consensus cartilage stem cell, cell sources that are utilized for cartilage transplantation in the clinic are limited to autologous chondrocytes. Techniques like microfracture are utilized to provide access for endogenous bone-marrow mesenchymal stem cells (MSCs) to cartilage; however, the MSCs at best give rise to functionally suboptimal fibrocartilage, due to its lack of t native collagen ECM. Tissue engineering approaches have explored alternative stem cell sources including adipose-derived stem cells (ADSCs) [33], clonal blood marrow precursors [34], and nasal cartilage-derived progenitor cells [35]; however, the persistent paucity of cells and a rapid loss of stemness upon expansion has been a bottleneck for these cell sources as well.

Previous studies have suggested that juvenile chondrocytes could be an attractive cell source for cartilage generation owing to their faster proliferation and expansion, but their widespread use is limited by donor availability. Patient-specific hiPSCs can be generated from readily available cells including blood and skin cells, thereby overcoming the major bottleneck of the paucity of cells and donor availability. The aim of these studies was to provide a detailed side-by-side phenotypic as well as molecular comparison of adult, juvenile, and human iPSC-derived chondrocytes (hiChondrocytes). hiChondrocytes were generated from hiPSCs using our previously published methodology that led to an efficient and homogeneous differentiation [18]. hiChondrocytes exhibited a higher proliferation rate similar to juvenile chondrocytes that would be advantageous in their expansion for research or therapeutic applications.

Detailed analyses of global gene expression patterns of hiChondrocytes, juvenile chondrocytes, and adult chondrocytes allowed us to dissect the common and distinct molecular factors between the chondrocyte populations. We expected variability in the chondrocyte populations isolated from different donors (i.e., juvenile and adult chondrocytes as well as different hiChondrocyte lines). Studies such as those reported here have the caveat that they are based on a small subset of available samples. Analyses of large cohorts of ‘normal’ juvenile or adult chondrocytes are a challenge due to both the limited availability of these samples and the limited cell expansion possible before these cells dedifferentiate in culture. These studies, however, are useful in identifying putative differential ‘factors’ and their functions that can be potentially utilized for modulating and rejuvenating available chondrocyte populations. For example, our analyses report novel ECM genes (*CHRDL1* and *MFAP4*) that are enriched in both juvenile and hiChondrocytes as compared to the adult chondrocytes. One of these ECM genes, *CHRDL1*, is stimulatory for resident MSCs [21] and could therefore contribute to a superior regeneration in vivo.

Another surprising find was that immune response pathways were differentially enriched both in hiChondrocytes and in juvenile chondrocytes. This led to the interesting finding that hiChondrocytes mimic juvenile chondrocytes in a milder response to proinflammatory cues compared to adult chondrocytes. Upon IL-1β stimulation, both hiChondrocytes and juvenile chondrocytes showed a lower upregulation of inflammatory (like *CCL2* and *IL6*) and catabolic (*MMP3* and *ADAMTS4*) genes than adult chondrocytes. In addition, hiChondrocytes and juvenile chondrocytes were also resistant to dedifferentiation compared to adult chondrocytes since they maintained the expression of chondrogenic genes, *COL2A* and *SOX9*, in the presence of IL-1β, while these chondrogenic genes were rapidly downregulated in adult chondrocytes.

CD24 is a small, glycosyl-phosphatidylinositol (GPI)-anchored cell-surface protein that is heavily glycosylated in a highly tissue and context-dependent manner. CD24 lacks an intracellular domain but is known to interact with multiple partners leading to different signaling outcomes in neural and hematopoietic tissues, including effects on proliferation, differentiation, and inflammation [36, 37]. CD24 was previously found to be enriched in juvenile chondrocytes compared to adult chondrocytes and was reported by us to be protective against IL-1β [26]. In the present studies, we have also identified its effect on chondrocyte proliferation since a loss of CD24 led to reduced cell proliferation. CD24 is therefore one of the factors that contribute to the dual advantage of both higher proliferation and resistance to IL-1β in hiChondrocytes and juvenile chondrocytes, characteristics that could potentially translate into superior cartilage regeneration in vivo.

Upon encapsulation of chondrocytes in chondroitin sulfate containing polyethylene glycol (CS-PEG)-based biomimetic hydrogels, transplantation in vivo in immune-deficient mice, and harvesting after 4 weeks, the cartilage constructs generated by hiChondrocytes and juvenile chondrocytes showed high expression of distinct chondrogenic genes (i.e., *SOX9*, *COL2A1*, *AGAN*) and minimal or no expression of the hypertrophy marker COl10A1. These data are consistent with previously established quantitative functional methods to distinguish juvenile and adult chondrocytes with characteristics such as increased ECM production by the juvenile chondrocytes compared to adult chondrocytes [20]. Importantly, these data suggest that hiChondrocytes are similar to juvenile chondrocytes in their high proliferative capacity and generation of cartilage constructs in vivo in hydrogel scaffolds. Although our studies had to be carried out in immune-deficient mice to prevent outright rejection of the human cells in mice, it can be imagined that the resistance to IL-1β would likely be a further big advantage in the utility of hiChondrocytes in a clinical setting in diseases like osteoarthritis or rheumatoid arthritis where end-stage chronic inflammation is a major component.

## Conclusions

This study provides a detailed fundamental characterization of hiChondrocytes in comparison to juvenile and adult chondrocytes phenotypically, molecularly, and functionally. Human iPSC-derived chondrocytes mimic juvenile chondrocytes in the multiple advantages of abundance, high proliferation, unique ECM generation, and resistance to the inflammatory cytokine IL-1β. These characteristics establish hiChondrocytes as an attractive, abundant, and autologous cell source for cartilage engineering. In addition, this study is fundamentally interesting because it provides an exemplar for how the developmental immaturity of hiPSC-derived cells can be an advantage for generating a tissue like cartilage with poor regenerative capability in adulthood in contrast to tissues like heart and brain, where such a developmental immaturity is a bottleneck.

## Additional file

Additional file 1: Figure S1. Global gene expression patterns in hiChondrocytes and juvenile chondrocytes are distinct from adult chondrocytes: (a) scheme for a step-wise hiChondrocyte differentiation from hiPSC using defined growth factors, (b) cell proliferation kinetics of hiChondrocytes, adult chondrocytes, and juvenile chondrocytes over 24, 48, and 96 hours, (c)heatmap depicting global gene expression patterns of adult, juvenile, hiChondrocytes, and hiPSC. **Figure S2**. Correlation-matrices generated for the global gene expression profiles for all the samples showing clustering of the chondrocyte samples such that the juvenile, adult, or hiChondrocyte populations could not be distinguished from each other while being distinct from iPSC: (a)while hiC1 showed a higher correlation with the adult chondrocyte samples A1 and A2 in comparison with the juvenile samples J1 and J3, hiC2 showed a similar correlation with A1, A2, J1, and J3;(b)inclusion of J2 and A3 further highlighted the variability between the donors, both adult and juvenile, showing that at the global level it is not possible to distinguish hiChondrocytes from juvenile or adult chondrocytes. **Figure S3**. List of 71 common genes that are at least 2-fold enriched in both hiChondrocytes and juvenile chondrocytes as compared to adult chondrocytes divided into genes that are higher in juvenile chondrocytes or hiChondrocytes. **Figure S4**. Real-time PCR indicating gene expression levels of *LBD2, EPHA5, ASPN, CDH10, NEDD9, and CD34 *in hiChondrocytes and juvenile chondrocyte samples relative to adult chondrocytes.** Figure S5**. Real-time PCR indicating that gene expression levels of *SOX9, COL2A1, and ACAN* in hiChondrocytes are comparable to juvenile as well as adult chondrocytes. **Figure S6**. (a)relative CD24 gene expression and (b)flow cytometry identifying CD24+cells in hiChondrocytes in control (shNTC) and upon CD24 knockdown (shCD24). **Figure S7**. Annexin V expression at a single cell level measured by FACS to identify the percentage of cells undergoing apoptosis in the presence (shNTC) or absence of CD24 (shCD24). (PDF 1419 kb)
