# Supplementary figures and images for: Human iPSC-derived chondrocytes mimic juvenile chondrocyte function for the dual advantage of increased proliferation and resistance to IL-1β
Source: Stem Cell Res Ther. 2017 Nov 2;8:244. doi: 10.1186/s13287-017-0696-x (PMC5667438; doi:10.1186/s13287-017-0696-x)

Figure S1

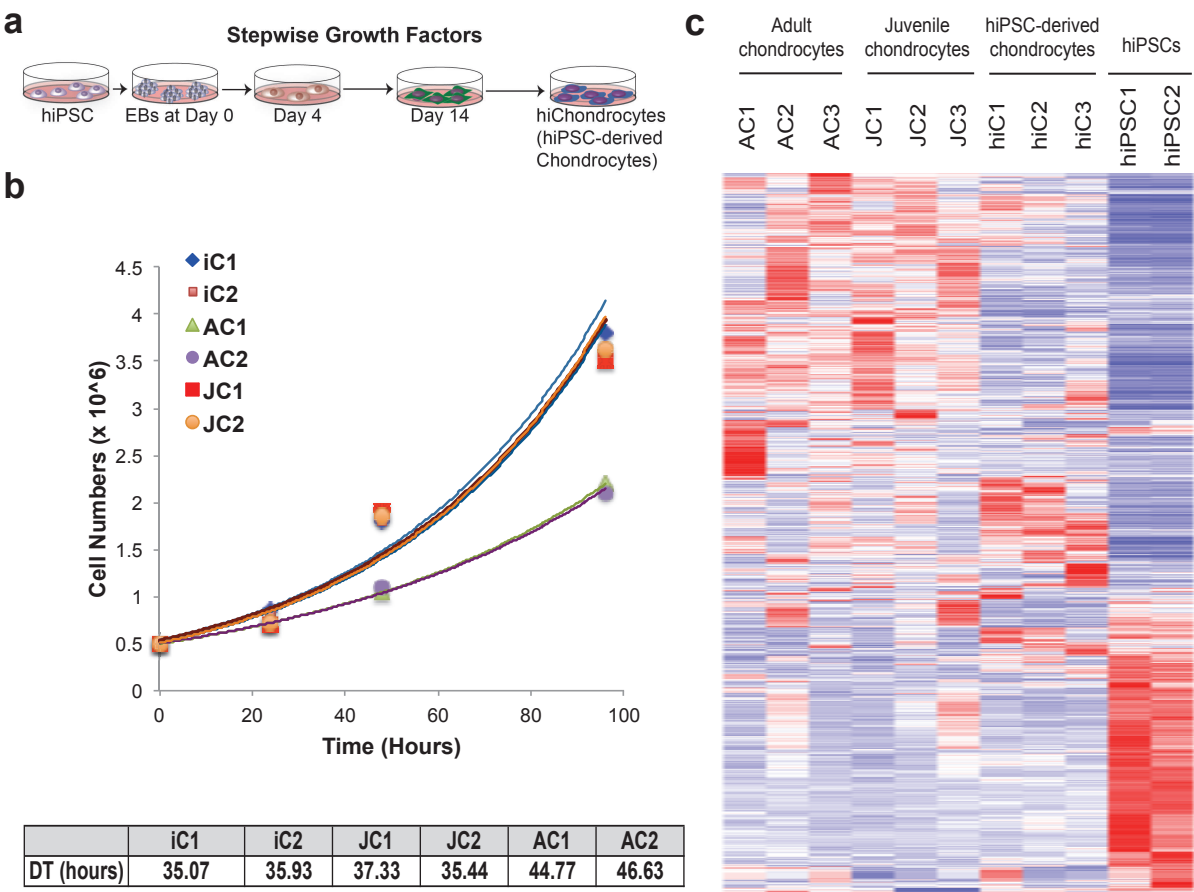

Figure S2

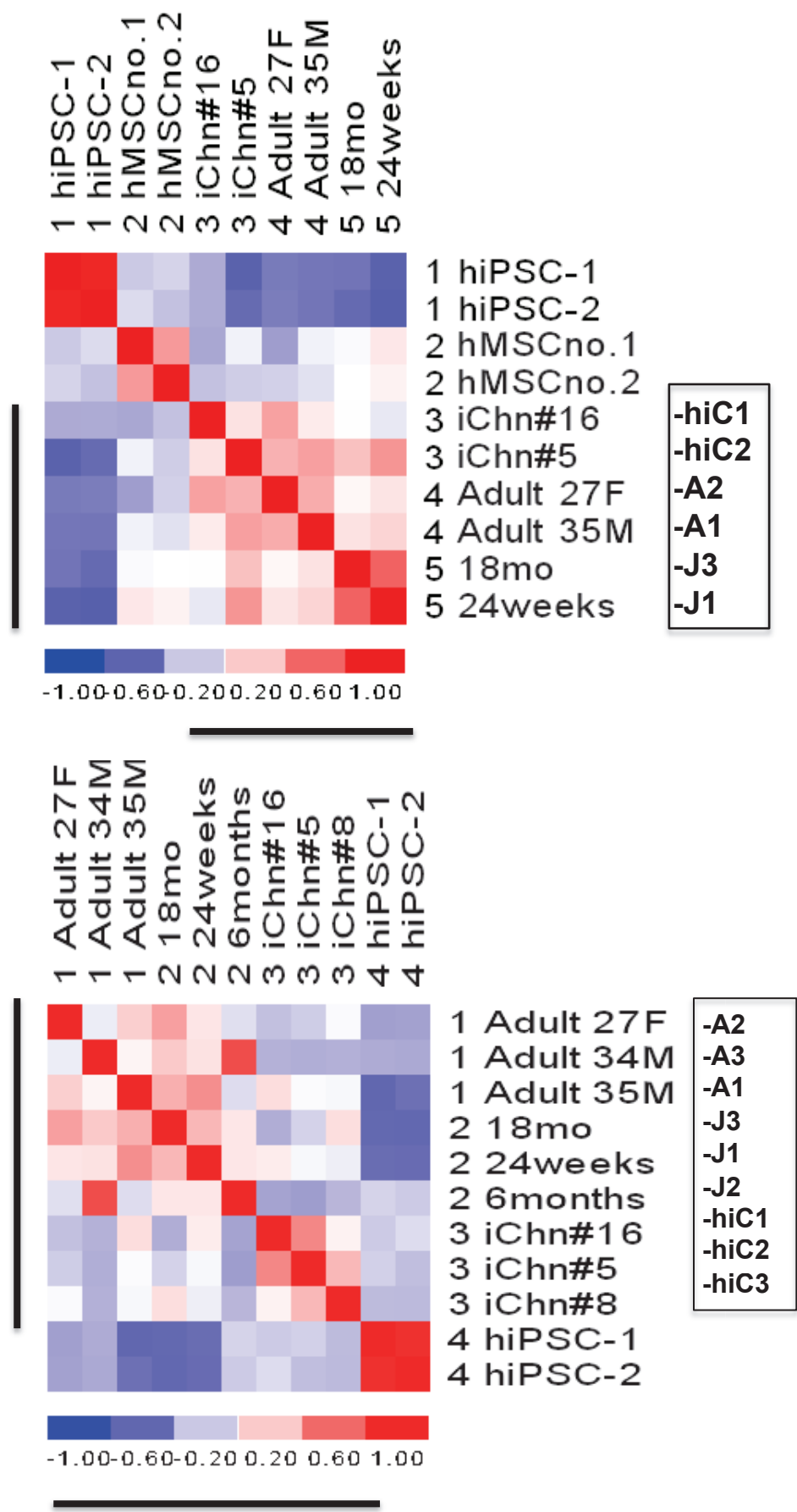

Figure S3

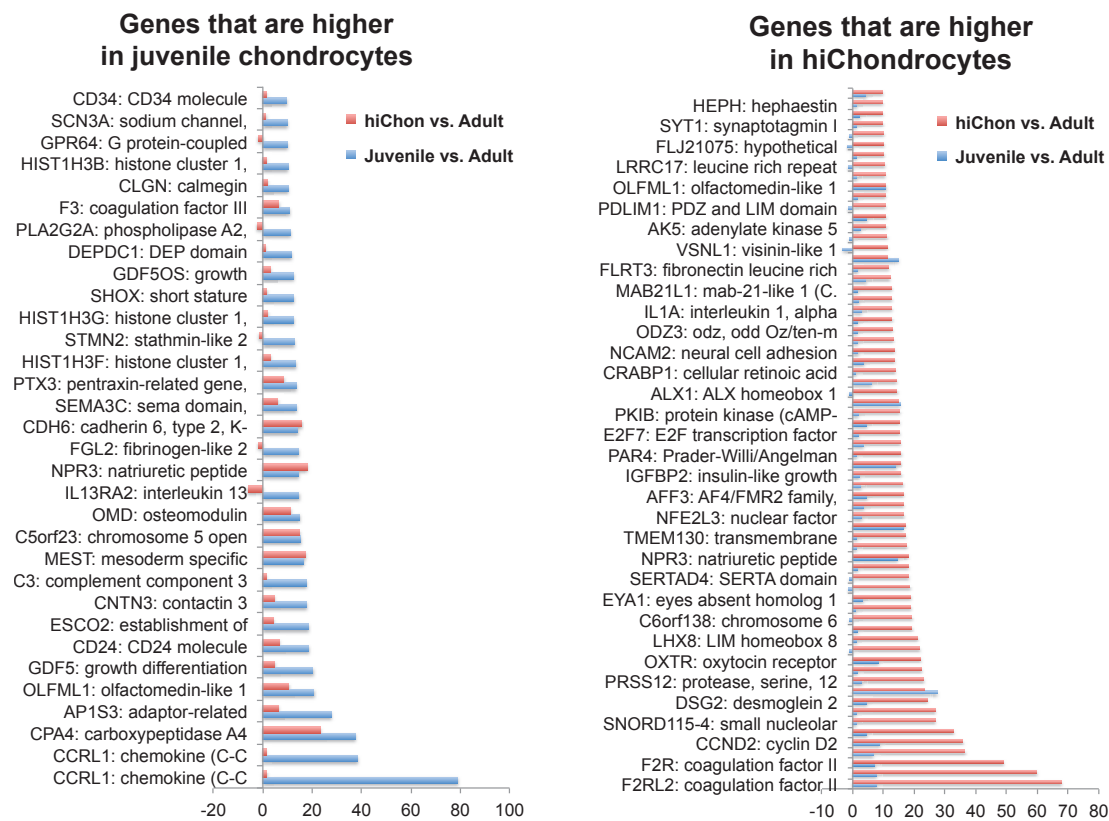

Figure S4

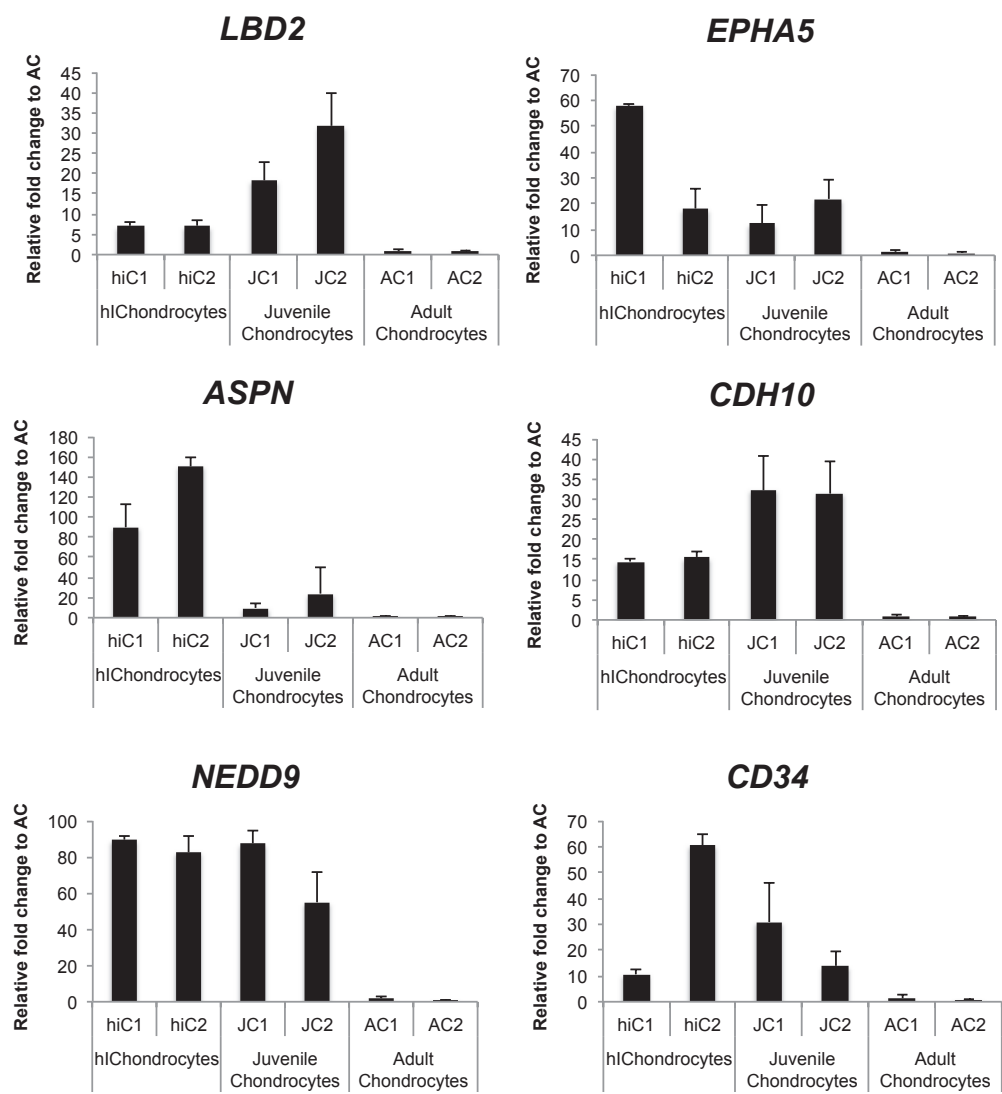

Figure S5

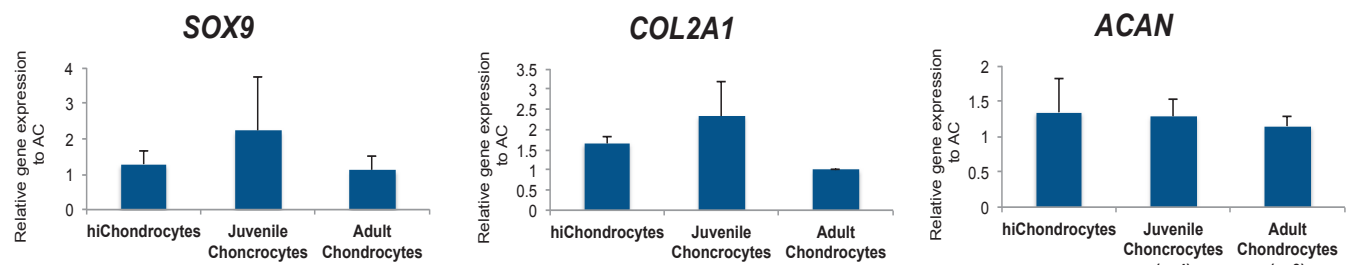

Figure S6

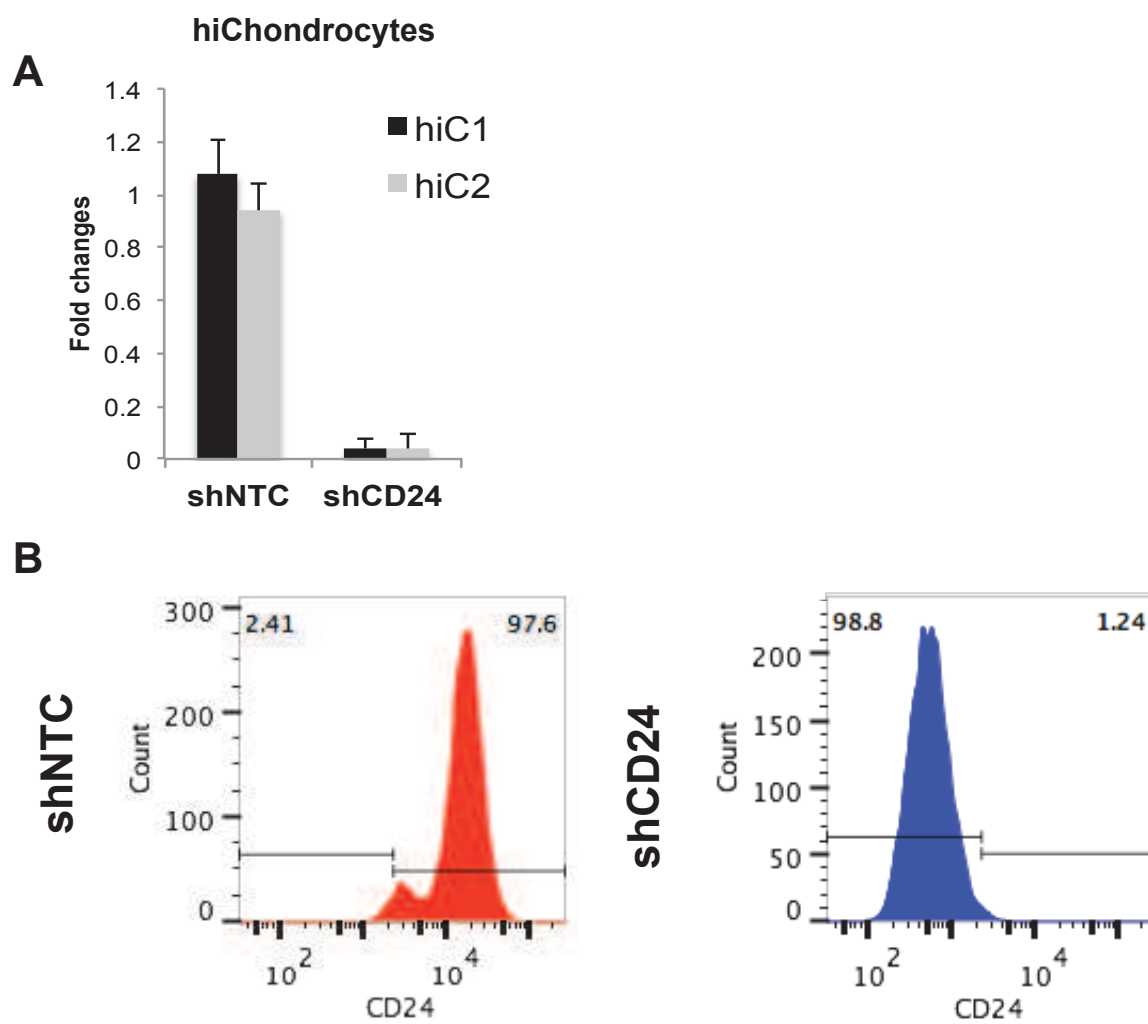

Figure S7

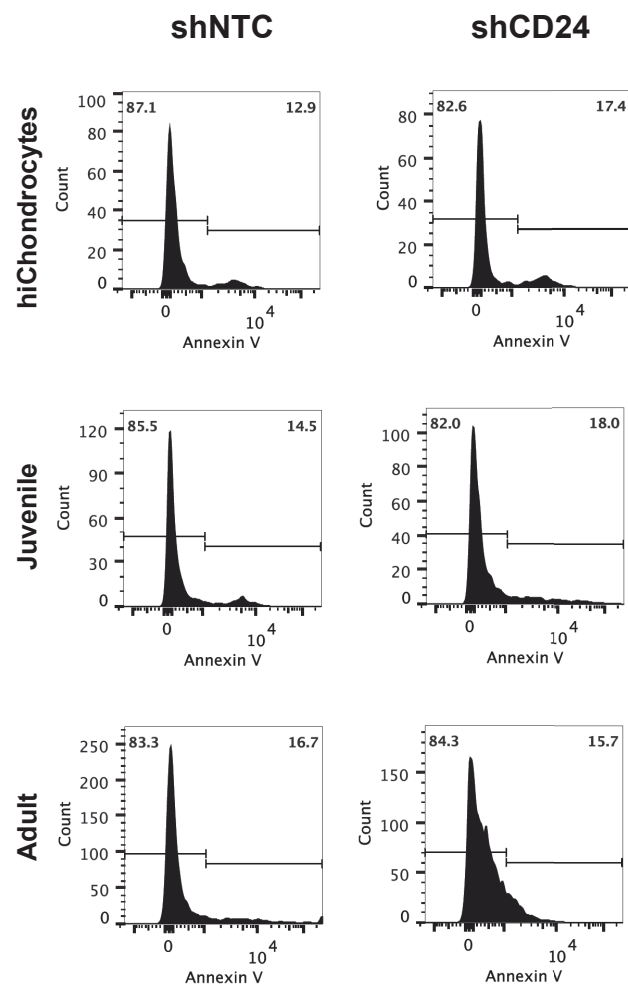

Supplement: Additional file 1: Figure S1. — Global gene expression patterns in hiChondrocytes and juvenile chondrocytes are distinct from adult chondrocytes: (a) scheme for a step-wise hiChondrocyte differentiation from hiPSC using defined growth factors, (b) cell proliferation kinetics of hiChondrocytes, adult chondrocytes, and juvenile chondrocytes over 24, 48, and 96 hours, (c)heatmap depicting global gene expression patterns of adult, juvenile, hiChondrocytes, and hiPSC. Figure S2. Correlation-matrices generated for the global gene expression profiles for all the samples showing clustering of the chondrocyte samples such that the juvenile, adult, or hiChondrocyte populations could not be distinguished from each other while being distinct from iPSC: (a)while hiC1 showed a higher correlation with the adult chondrocyte samples A1 and A2 in comparison with the juvenile samples J1 and J3, hiC2 showed a similar correlation with A1, A2, J1, and J3;(b)inclusion of J2 and A3 further highlighted the variability between the donors, both adult and juvenile, showing that at the global level it is not possible to distinguish hiChondrocytes from juvenile or adult chondrocytes. Figure S3. List of 71 common genes that are at least 2-fold enriched in both hiChondrocytes and juvenile chondrocytes as compared to adult chondrocytes divided into genes that are higher in juvenile chondrocytes or hiChondrocytes. Figure S4. Real-time PCR indicating gene expression levels of LBD2, EPHA5, ASPN, CDH10, NEDD9, and CD34 in hiChondrocytes and juvenile chondrocyte samples relative to adult chondrocytes. Figure S5. Real-time PCR indicating that gene expression levels of SOX9, COL2A1, and ACAN in hiChondrocytes are comparable to juvenile as well as adult chondrocytes. Figure S6. (a)relative CD24 gene expression and (b)flow cytometry identifying CD24+cells in hiChondrocytes in control (shNTC) and upon CD24 knockdown (shCD24). Figure S7. Annexin V expression at a single cell level measured by FACS to identify the percentage of cells undergo [file 13287_2017_696_MOESM1_ESM.pdf]
